# Atypical Mesenchymal Stromal Cell Responses to Topographic Modifications of Titanium Biomaterials Indicate Cytoskeletal- and Genetic Plasticity-Based Heterogeneity of Cells

**DOI:** 10.1155/2019/5214501

**Published:** 2019-07-01

**Authors:** Mohammad R. Khan, Nicola Mordan, Mohamed Parkar, Vehid Salih, Nikolaos Donos, Peter M. Brett

**Affiliations:** ^1^Biomaterials and Tissue Engineering, UCL Eastman Dental Institute, University College London, 256 Gray's Inn Road, London WC1X 8LD, UK; ^2^Faculty of Medicine & Dentistry, University of Plymouth, Drake Circus, Plymouth PL4 8AA, UK; ^3^Institute of Dentistry, Queen Mary University of London, Turner Street, London E1 2AD, UK; ^4^University of Surrey, Guildford, Surrey GU2 7XH, UK

## Abstract

Titanium (Ti) is widely used as a biomaterial for endosseous implants due to its relatively inert surface oxide layer that enables implanted devices the ability of assembling tissue reparative components that culminate in osseointegration. Topographic modifications in the form of micro- and nanoscaled structures significantly promote osseointegration and enhance the osteogenic differentiation of adult mesenchymal stromal cells (MSCs). While the biological mechanisms central to the differential responses of tissues and cells to Ti surface modifications remain unknown, adhesion and morphological adaptation are amongst the earliest events at the cell-biomaterial interface that are highly influenced by surface topography and profoundly impact the regulation of stem cell fate determination. This study correlated the effects of Ti topographic modifications on adhesion and morphological adaptation of human MSCs with phenotypic change. The results showed that modified Ti topographies precluded the adhesion of a subset of MSCs while incurring distinct morphological constraints on adherent cells. These effects anomalously corresponded with a differential expression of stem cell pluripotency and Wnt signalling-associated markers on both modified surfaces while additionally differing between hydrophobic and hydrophilic surface modifications—though extent of osteogenic differentiation induced by both modified topographies yielded similarly significant higher levels of cellular mineralisation in contrast to polished Ti. These results suggest that in the absence of deposited proteins and soluble factors, both modified topographies incur the selective adhesion of a subpopulation of progenitors with relatively higher cytoskeletal plasticity. While the presence of deposited proteins and soluble factors does not significantly affect adherence of cells, nanotopographic modifications enhance expression of pluripotency markers in proliferative conditions, which are conversely overridden by both modified topographies in osteogenic inductive conditions. Further deciphering the mechanisms underlying cellular selectivity and Ti topographic responsiveness will improve our understanding of stem cell heterogeneity and advance the potential of MSCs in regenerative medicine.

## 1. Introduction

Titanium (Ti) is widely used as a biomaterial for dental and orthopaedic endosseous applications due to its mechanical properties and high biocompatibility. Highly reactive in its elemental state, Ti spontaneously forms a relatively bioinert surface oxide layer that enables endosseous devices the ability of assembling tissue reparative components that culminate in osseointegration, an uninterrupted contact between the surface of an implant and adjacent bone [1, 2]. Enhancements to the quantifiable parameters of osseointegration that include increased implant-bone contact, denser accrual of peri-implant bone, and a stronger mechanical interlock have been widely reported for Ti implants modified to a moderately rough topography by sand blasting and acid etching (SLA) [3–9]. An additional modification at the nanotopographic scale that renders the SLA surface hydrophilic due to increased surface-free energy by preventing adsorption of hydrocarbons has more recently been reported to further promote clinically graded osseointegration compared to the hydrophobic SLA in human and animal studies [10–13]. Experimental evidence implicates an earlier induction of osteogenic and angiogenic responses coupled with the inhibition of bone remodelling and inflammation as primary factors contributing to the temporal enhancement of osseointegration with modified SLA (modSLA) [14–17].

The *in vitro* study of cellular responses to the SLA and modSLA collectively suggests a novel promotion of osteogenic differentiation, maturation, and function [18–20]. These effects seem particularly evident for bone marrow-derived mesenchymal stromal cells (MSCs) that are multipotent clonogenic cells expressing a highly specific repertoire of cell surface markers and are capable of trilineage differentiation to osteogenic, adipogenic, and chondrogenic phenotypes [21]. In addition to the potential therapeutic benefits and a proposed role in wound repair, MSCs are an important model for *in vitro* cell and tissue developmental studies. Our observations of MSC responses to the SLA and modSLA have correlated a relatively earlier induction of cell growth, survival, and osteogenic differentiation-related genetic markers with an enhancement in extracellular matrix elaboration and calcification, with a further correlation drawn with the concomitant downregulation of cell signalling molecules believed to confer MSCs with multipotentiality [20, 22, 23].

Furthermore, due to a highly sensitive and responsive mechano-transduction apparatus, MSC fate determination is significantly dependent on adhesion and morphological cues [24]. Experimental evidence pertaining to MSCs and osteoblasts variably reports differences in cellular attachment but consistently indicates distinct morphological differences between cells adhering to the polished and modified Ti surfaces [18, 25–28]. More recent investigations have implicated the GTPase RhoA and ROCK signalling with increased osteoblastic cell differentiation on the SLA and modSLA due to morphological constraints enacting on various cytoskeletal components [29–31]. These constraints affect the structuring of actin and myosin fibres during cell adhesion, which activates intracellular signalling related to the induction of osteogenic differentiation [32]. Importantly, however, while the cellular mechanisms correlating MSC adhesion and morphological adaptation to topographical cues of the SLA and modSLA have been defined to some extent, additional biological factors such as protein deposition and soluble molecules in the immediate microenvironment that may significantly influence surface-related effects on intracellular signalling and phenotypic maturation are yet to be known [30].

Accordingly, the present study compared adhesion and morphological adaptation of human MSCs to Ti biomaterials with different topographic modifications and correlated these differences with the expression of stemness-related pluripotency markers and osteogenic differentiation. It proposed that adhesion and morphological adaptation to modified Ti surfaces would differentially affect the regulation of expression of genes imparting MSCs with stem cell multipotency in conjunction with chemical cues from growth and osteogenic culture media in determining stem cell fate. In order to examine this hypothesis, adult human bone marrow-derived MSCs were seeded on tissue culture polystyrene (TCP), polished (P), hydrophobic SLA and hydrophilic SLA (modSLA) surfaces in a serum-free culture medium. Cell labelling assays were used to initially estimate the number of adherent cells at 1, 3, and 24 hours postcontact and thereafter at 15 min intervals within the first hour of cell-substrate contact. Samples from the cell adhesion experiments were examined with back-scattered electron microscopy to confirm the relative number of adherent cells and then sputter coated for scanning electron microscopy to image morphological differences between MSCs on the different Ti surfaces. In addition to an osteogenic mineralisation assay, a comparison of the expression of stemness-related pluripotency genetic markers was performed with real-time polymerase chain reactions (RT-PCR) miniarrays with further four Wnt signalling-associated markers that our experience has shown to have significant surface- and microenvironment-dependent effects.

## 2. Materials and Methods

### 2.1. Cell Culture

Human bone marrow-derived multipotent stromal cells (MSCs) were acquired from the Institute for Regenerative Medicine, Texas A&M Health Science Centre, College of Medicine (USA). Three donor cell lines (Caucasian; male; 20-30-year age group) were used in this study, which had been isolated from bone marrow aspirates using Ficoll density centrifugation and characterised for single cell-derived colony formations, trilineage differentiation, and expression of a repertoire of MSC-specific cell surface markers. For this study, the MSCs were cultured according to the parameters suggested by [33]. Cells were expanded in T150 culture flasks (Nunc, Denmark) at a low seeding density of 100 cells per cm^2^ in growth medium (GM) that comprised minimal essential medium alpha (Gibco, UK) supplemented with 10% lot selected fetal bovine serum (Gibco, UK), 1% antibiotics (penicillin/streptomycin; PAA Laboratories, UK), and 0.1% fungicide (amphotericin B; Gibco, UK) in humidified conditions (37°C and 5% CO_2_). Cultures underwent two medium changes in seven days and were harvested for experimentation on attaining 80% confluence with 0.05%/0.002% Trypsin/EDTA in Ca^+2^/Mg^+2^-free PBS (PAA Laboratories, UK). Experiments were performed with cells that had undergone a maximum of three passages.

### 2.2. Titanium Biomaterials

The Ti biomaterials were supplied by Institut Straumann AG (Basel, Switzerland). Three modified Ti implant surface topographies were used in this study: a polished (P), rough hydrophobic sand blasted acid etched (SLA), and rough hydrophilic (modSLA). These topographies were fabricated on discs of 1.8 cm^2^ surface area (diameter = 15 mm). The methods of fabricating these topographies as well as the physical and chemical characterisations have been outlined in [20, 27, 34]. The P and SLA discs were passivated prior to use by immersing in 10% *v* / *v* nitric acid in deionised water, air-dried, and sterilised with UV irradiation. The modSLA discs were supplied in sterile saline in glass vials. Tissue culture plastic (TCP) was used as a control surface.

### 2.3. CyQuant Nucleic Acid Stain Quantification

The CyQuant GR assay (Sigma-Aldrich, UK) was used to determine the number of attached MSCs at 1, 3, and 24 h post seeding on TCP, P, SLA, and modSLA discs. The kit consists of a fluorescent dye that exhibits enhanced fluorescence on intercalating with double-stranded nucleic acids of ruptured cells and was used according to the manufacturer's instructions. For this experiment, human MSCs (*N* = 2) were expanded as described above. Cells were detached from culture flasks and then seeded at a density of 1 × 10^4^ cells per surface in 1 ml of serum-free basal medium (alpha-MEM, Gibco, UK). Adherence of MSCs to each surface was evaluated with three replicates (*n* = 3) per cell line. At each time point, samples were gently washed twice with PBS and lysed by ×3 freeze-thaw cycle that alternated between -80°C and room temperature, during which the samples in 24-well plates (Nunc) were firmly enclosed using sealing film (Parafilm M, Sigma-Aldrich, UK). A working solution of dye was then added at 150 *μ*l per well for 10 min at 4°C in the dark. A 100 *μ*l volume of homogenate per sample was transferred to an opaque 96-well plate (Nunc) for fluorescence intensity measurement at excitation 530 nm and emission 590 nm (Fluoroscan, Tecan, Switzerland). Total cell numbers were determined by interpolating fluorescent intensities of samples from a standard curve.

### 2.4. Calcein-AM Cell Label Assay

The Calcein-AM cell label (Vybrant Cell Adhesion kit, Sigma-Aldrich, UK) was used to determine the number of cells adherent to the P, SLA, and modSLA surfaces at 5, 15, 30, and 60 min post seeding. For this experiment, expanded MSCs from three cell lines (*N* = 3) were trypsinised from culture flasks and normalised to a density of 5 × 10^6^ cells per ml in growth medium in 50 ml tubes (Falcon, UK). The cell suspension was incubated with 5 *μ*M of Calcein-AM dye in serum-free alpha-MEM for 30 min in humidified conditions. Labelled cells were washed three times by centrifuging at 500 x *g* for 5 min in PBS and resuspended in serum-free alpha-MEM for a trypan blue-based viable cell count. Viable cells in serum-free alpha-MEM were seeded at a density of 3 × 10^4^ cells per disc in triplicate. This higher cell density was used compared to the previous experiment to overcome any loss of detection of fluorescence due to low adherence of cells resulting from extremely short durations of contact with the Ti surfaces. At each time point, nonadherent cells were removed by gentle pipetting and samples were gently washed three times with PBS. Fluorescence intensity was measured directly at excitation 530 nm and emission 590 nm in a fluorometer (FluoroScan, UK). Cell-related fluorescence was determined by subtracting background fluorescence readings of corresponding clean surfaces and then interpolating the difference from a standard curve. Additional samples for 3 and 24 h contact were prepared for morphological assessments.

### 2.5. Electron Microscopy

Scanning electron microscopy (SEM) was conducted on a Cambridge Stereoscan S90B (Cambridge Instruments, UK). Samples from the Calcein-AM-based experiment were fixed immediately after fluorescence measurements in 3% glutaraldehyde in 0.1 M sodium cacodylate buffer (pH 7.3) (Agar Scientific, Stansted, Essex, UK) at 4°C overnight. Samples were later dehydrated in a graded series of alcohols (50%, 70%, 90%, and twice with 100%) and critically dried in hexamethyldisilazane (TAAB Laboratories Ltd., Reading, Berkshire, UK) for 5 min at room temperature. Samples were imaged with back-scattered SEM, which differentiates between an inorganic bright background and opaque/dark organic cells. A manual count was performed to verify the relative difference in the number of adherent MSCs on each surface. These samples were sputter coated with gold/palladium using a Polaron E5000 Sputter Coater (Quorum Technologies Ltd., Newhaven, East Sussex, UK) and visualised with SEM.

### 2.6. Gene Expression of Wnt Signalling Molecules

Real-time PCR was used to examine differences in the expression of Wnt signalling molecule osteoblastogenesis-related Wnt5a, osteoblastic marker Growth Differentiation Factor type 15 (GDF15), proliferative marker MKI67, and osteogenic marker osteocalcin (bone gamma-carboxyglutamic acid-containing protein). For this experiment, MSCs isolated from three donors (*N* = 3) were experimented in three technical replicates per donor (*n* = 3). The culture expanded MSCs (*N* = 3, *n* = 3) were seeded at a density of 3 × 10^4^ cells on TCP, P, SLA, and modSLA in growth medium (GM) and osteogenic medium (OM) for 24 h. Samples were washed twice with PBS and lysed *in situ* with buffer RLT for RNA extraction with the RNeasy Mini kit (Qiagen, USA) according to the manufacturer's instructions. Total RNA was eluted with 40 *μ*l of RNAse-free water per column and nucleic acid integrity quantified by spectrophotometry (Tecan NanoDrop, Switzerland) at excitation 260 nm and emission 280 nm. Then, 400 ng of RNA per sample was converted to 100 *μ*l cDNA by first-strand synthesis reactions with the High-Capacity Reverse Transcription Kit (Applied Biosystems, USA) according to the manufacturer's instructions. Real-time PCR reactions were performed in 25 *μ*l volume reactions with 2.5 *μ*l (10 ng of RNA equivalent) of cDNA per reaction in a 7300 Real-Time PCR Thermocycler (Applied Biosystems, USA). The probes used were Hs00998537_m1 for Wnt5a; Hs00171132_m1 for GDF15; Hs01032439_m1 for MKI67; and Hs01587814_g1 for OC/BGLAP (all from Applied Biosystems, USA). The Ct values of markers were normalised to the GAPDH housekeeping gene and calibrated to ΔCt values of cells in suspension (at time zero) to obtain relative fold value of expression.

### 2.7. Gene Expression of MSC Stemness Molecules

Changes in the expression of stem cell multipotency genes were evaluated with the Taqman Real-Time PCR Stem Cell Pluripotency Microfluidic Arrays (Applied Biosystems; catalogue number 4385344). These are 384-well plates (8 rows, 48 columns) that contain four groups of 96 genes that represent different categories related to stem cell biology. These are (i) expression in undifferentiated cells, (ii) maintenance of pluripotency, (iii) correlation to stemness, and (iv) differentiation markers, in addition to endogenous controls. For this experiment, MSCs were seeded at a density of 3 × 10^4^ cells on P, SLA, and modSLA in growth medium (GM) as well as osteogenic inductive medium (OM) for 24 h. Samples were washed twice with PBS and lysed *in situ* with buffer RLT for RNA extraction with the RNeasy Mini kit (Qiagen, UK) according to the manufacturer's instructions. Total RNA was eluted with 40 *μ*l of RNAse-free water per column and nucleic acid integrity quantified by spectrophotometry (Tecan NanoDrop, Switzerland) at excitation 260 nm and emission 280 nm. Then, 400 ng of RNA per sample was converted to 100 *μ*l cDNA by first-strand synthesis reactions with the High-Capacity Reverse Transcription Kit (Applied Biosystems, USA) according to the manufacturer's instructions. The arrays were run in an Applied Biosystems 9700 Real-Time PCR thermocycler at the University College London Institute of Child Health (London, UK). Fold values of changes in gene expression were evaluated with the 2^-ΔΔCt^ formula. 18S ribosomal RNA was used as the internal control to obtain delta Ct values. Delta Ct values of MSCs in suspension prior to plating cells on the surfaces were processed at the start of the experiment and used as a calibrator to obtain mean fold values of expression, which were plotted in a heatmap using the “R” software for statistical computing (http://www.r-project.org/). Euclidean distance was used to cluster similarly expressed genes into groups.

### 2.8. Osteogenic Differentiation

Osteogenic differentiation was performed by culturing MSCs in 24-well plates for 7 days with osteogenic inductive medium (OM), comprising Dulbecco's modified Eagle's medium low glucose (Gibco) supplemented with 10% lot selected fetal calf serum (Gibco, UK), 1% antibiotics and 0.1% fungicide (Gibco, UK), 10 nM dexamethasone (water soluble; Sigma-Aldrich, UK), 10 mM *β*-glycerophosphate (Fisher Scientific, UK), and 50 *μ*M ascorbate-2-phosphate (Fluka, UK). Samples were washed twice with PBS and fixed in 10% formalin for 10-15 min before being stained with a 2% Alizarin Red S (Sigma-Aldrich, UK) solution in deionised water (pH 4.1-4.3) for 10 min. Samples were washed 4x with deionised water and air dried. Calcium-bound dye was eluted with 0.5 ml of 10% (*w* / *v*) cetylpyridinium chloride in 10 mM sodium phosphate buffer, pH 7.2 (all Sigma-Aldrich, UK). Spectrophotometric absorbance of elutant was measured at 562 nm (Tecan, Switzerland).

### 2.9. Statistics

The cell-based assays were performed with primary human MSCs from three different donors (*N* = 3). Each donor was tested in three experimental replications (*n* = 3). Data was analysed with GraphPad Prism (GraphPad Inc., USA) using the ANOVA statistical test. The miniarray data was collated in “R” with Euclidean algorithm used to determine similarities in gene expression patterns.

## 3. Results

### 3.1. MSC Adhesion to Different Ti Surfaces

**Figure 1:**
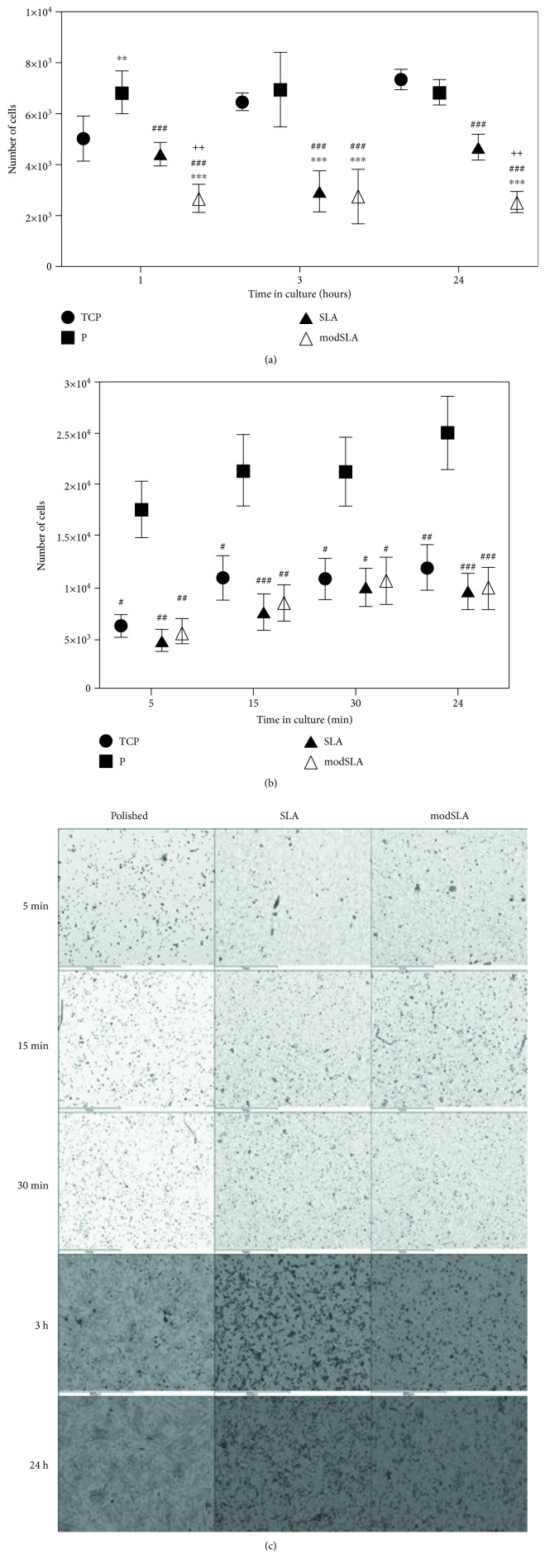
(a) Assessment of cell adhesion at 1, 3, and 24 h post contact demonstrated adherence at near-maximal numbers to each of the test surfaces by 1 h. Cell numbers increased slightly on tissue culture plastic but remained unchanged on the Ti surfaces. There was a significantly lesser number of cells on the rough compared to polished Ti surfaces. Shown are mean ± SD, *N* = donors = 2, *n* = replicates = 3. ^∗^*p* < 0.05, ^∗∗^*p* < 0.01, and ^∗∗∗^*p* < 0.001for tissue culture plastic compared to Ti surface. ^#^*p* < 0.05, ^##^*p* < 0.01, and ^###^*p* < 0.001, P compared to SLA or modSLA Ti surfaces. ^+^*p* < 0.05, ^++^*p* < 0.01, and ^+++^*p* < 0.001, SLA compared to modSLA Ti surfaces. (b) Assessment of MSC adhesion within the first hour of cell-substrate contact. The Calcein-AM cytoplasmic fluorescence labelling indicated a higher number of cells adherent to the polished Ti compared with TCP and both modified rough Ti surfaces. Shown are mean ± SD, *N* = 3, *n* = 3. ^#^*p* < 0.05, ^##^*p* < 0.01, and ^###^*p* < 0.001 for polished Ti compared to any surface. (c) Back-scattered scanning electron micrographs of Calcein-labelled samples from one MSC donor. Images of samples from the attachment assay confirmed the presence of a higher number of MSCs adherent to P compared to the modified rough Ti surfaces. Scale bar = 600 *μ*m.

Cell adhesion is the earliest event in cell-substrate interactions. It was hypothesised that the topographies of the polished, SLA, and modSLA Ti surfaces would differentially affect the adhesion of MSCs to these substrates. The results of the cell-substrate adhesion analysis performed using CyQuant GR nucleic acid staining of human MSCs in serum-free conditions are shown in Figure 1(a). Near-maximum MSC attachment to each of the four experimental surfaces had occurred by 1 h with detectable incremental increases taking place only on the TCP by 3 and 24 h. The polished Ti had a significantly higher number of adherent cells than SLA and modSLA (*p* < 0.001) at all time points. Between the rough surfaces, the hydrophobic SLA had a significantly higher number of adherent cells than the hydrophilic modSLA (*p* < 0.001) at 1 h and 24 h. Hence, using TCP as a control substrate for *in vitro* MSC culture and adhesion, differences between the number of cells adhering to the P, SLA, and modSLA surfaces were observed with a higher number of MSCs adherent to the P surface compared to both modified surfaces at 1, 3, and 24 h.

It was then decided to delineate differences in the earliest of time points hypothesising that contact durations of less than 1 h would further delineate the effects of substrates on cellular adhesion between different surfaces. This second analysis of human MSC adhesion in serum-free conditions was performed with a fluorescent cytoplasmic dye Calcein-AM and demonstrated a significantly higher number of cells adherent to the polished Ti compared to both rough surfaces (*p* < 0.01) at all four time points within the initial 1 h of contact. These results are shown in Figure 1(b).

To confirm the outcome of the Calcein-AM-based cell adhesion experiment, samples were visualised with back-scattered scanning electron microscopy (EM). These results are shown in Figure 1(c). A manual count of cells in the electron micrographs indicated quantitative differences with a higher number of adherent cells on the polished Ti surface compared to the rough surfaces at all points. An automated imaging software count of cells could not be performed due to an inability of software (ImageJ; https://imagej.nih.gov/ij/) to decipher cells from the topographical features of the rough surfaces.

### 3.2. MSC Morphology on Different Ti Surfaces

**Figure 2:**
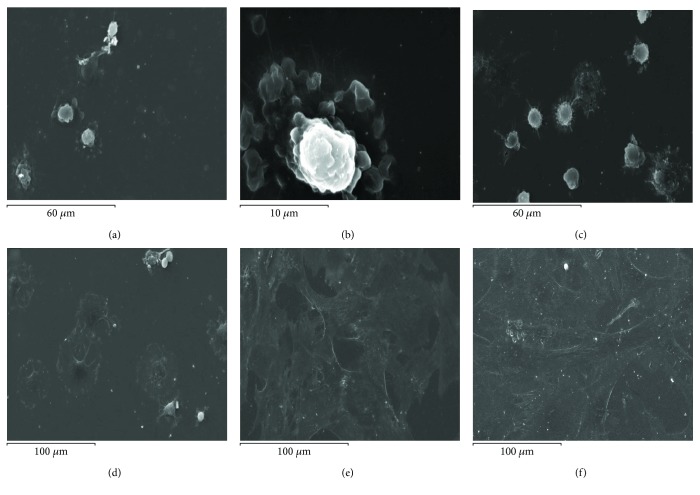
Morphological adaptation of MSCs on polished Ti. (a) Cells with a prominent nucleus and circumferentially spread cell membrane at 5 min post seeding. At (b) 15 min and (c) 30 min, cellular spreading had increased with some cells appearing comparatively rounded by this time. (d) By 1 h, most cells displayed a uniform circumferential spread over the surface. By (e) 3 h and (f) 24 h, cells had adapted an irregular and stretched polygonal morphology.

**Figure 3:**
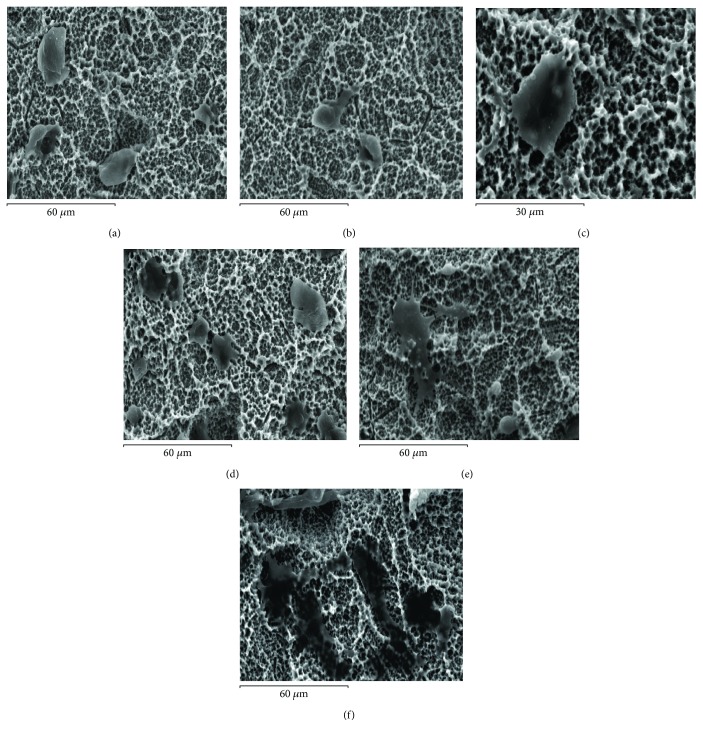
Morphological adaptation in MSCs on the SLA Ti surface. (a) At 5 min, cells appear round with membranous extensions emanating across the rough topography. At (b) 15 min and (c) 30 min, cells appear constrained. At (d) 1 h, cells displayed a slight shift from their previous circular form. At (e) 3 h, cells had adapted a stretched asymmetric morphology that progressed till (f) 24 h.

**Figure 4:**
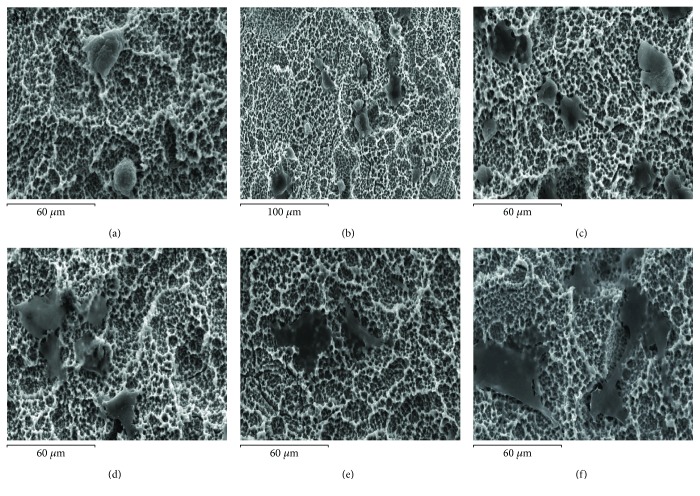
Morphological adaptation of MSCs on the modSLA Ti surface. MSCs exhibited similar morphological adaptations as when seeded on the SLA. (a–c) MSCs appeared very rounded at 5, 15, and 30 min post seeding. (d) MSCs displayed extensions emanating across the surface by 60 min and had (e) begun exhibiting a stretched asymmetric form by 3 h that continually progressed till 24 h (f).

Electron microscopy of sputter coated samples was then used to image the morphology of MSCs adherent to the different Ti surfaces at different time points. The morphology of cells after adhering to the Ti surfaces following different durations of contact is shown in Figures 2–4 for the polished, hydrophobic SLA, and hydrophilic modSLA, respectively. The MSCs appeared rounded with a prominent centrally located nucleus and concentrically distributed cytoplasm at 5–30 min post contact that continued to spread over the P surface by 1 h. By 3 to 24 h, cells seemed to have stretched over a large area of the substrate with a flattened morphology and without a prominent nucleus observable. This sequence of cellular spreading and morphological adaptation to the P surface is shown in Figure 2. Conversely, MSCs appeared to have a constrained morphology on both modified rough Ti surfaces. Adherent MSCs appeared predominantly rounded for the initial hour of contact with few cytoplasmic extensions emanating into the crevices of the rough topographies. MSCs progressed to exhibit an irregular “stellate” or star-shaped form at 3 h that did not change noticeably by 24 h on both rough surfaces. The sequence of morphological changes occurring on the SLA and modSLA is shown in Figures 3 and 4, respectively.

### 3.3. Gene Expression of Wnt Signalling-Related Markers

**Figure 5:**
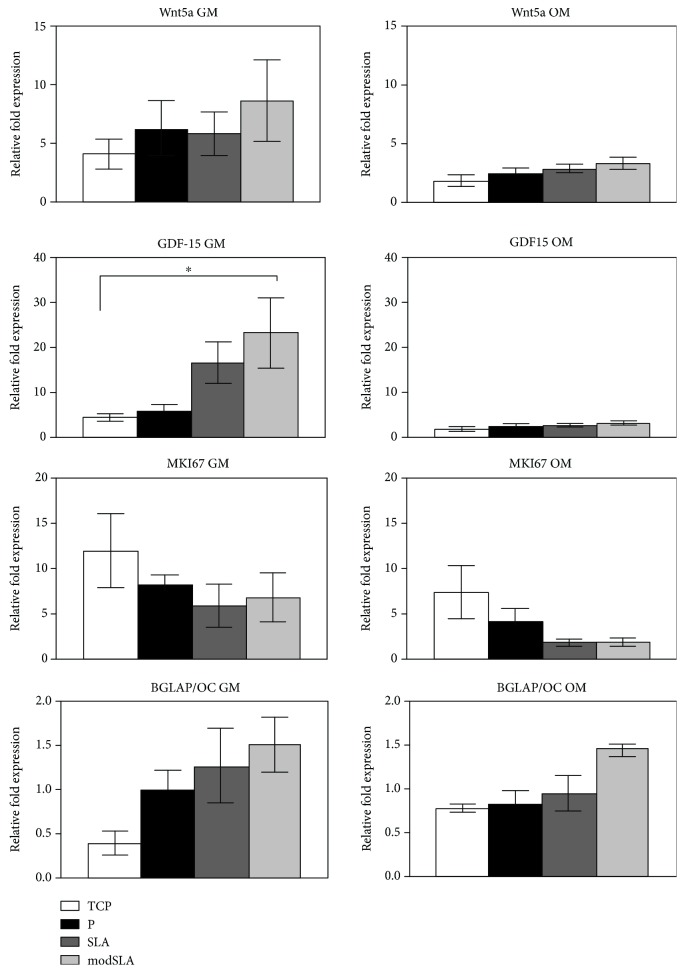
Assessment of Wnt signalling-associated genes with RT-PCR after 24 h. The Wnt molecules GDF-15 and Wnt5a were expressed by cells at higher levels in growth medium (GM) than in osteogenic medium (OM). Both molecules displayed a consistent trend of high expression on modSLA and SLA than P and TCP. Expression of proliferation marker MKI67 was markedly reduced by OM. It was expressed the highest on TCP than Ti surfaces in both medium types with rough surfaces further reducing expression in OM. OC was similar in both types of medium and displayed a trend of higher expression on modSLA. Shown are $\text{mean }\underset{¯}{+}\;1\text{SD}$; *N* = 3, *n* = 3. ^∗^*p* < 0.05.

The Wnt signalling pathway is associated with several cellular processes including survival, proliferation, and differentiation. The surface- and medium-dependent effects on the expression of Wnt signalling-associated molecules Wnt5a, GDF15, MKI67, and BGLAP/OC were evaluated in MSCs cultured in proliferative growth medium and osteogenic osteoinductive medium on P, SLA, and modSLA Ti surfaces. The results of this analysis are shown in Figure 5. Wnt5a, a marker associated with the promotion of osteogenic differentiation, was expressed several folds higher by MSCs in growth medium than in osteogenic medium though differences between the different surfaces were negligible. GDF-15, a marker associated with the promotion of cell proliferation, was expressed at higher levels in growth medium than in osteogenic medium, with both modified topographies SLA and modSLA inducing higher expression than TCP and P in proliferative GM. The expression of proliferation marker MKI67 was markedly reduced in osteogenic than growth medium with a higher expression on TCP than Ti surfaces. Osteocalcin, a calcium and hydroxyapatite binding protein, was expressed at similar levels in both types of media with relatively increased expression on Ti surfaces. Hence, Wnt signalling-related gene expression differed in MSCs cultured in the two different medium conditions, which influenced surface-dependent effects on cells.

### 3.4. Gene Expression of Pluripotency Stem Cell Markers

**Figure 6:**
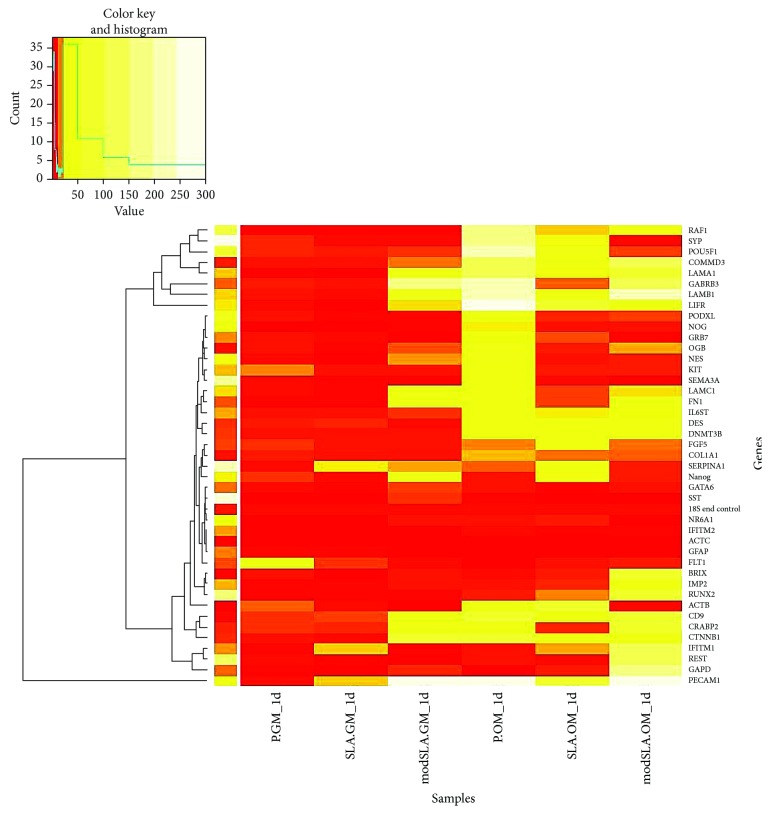
Analysis of pluripotency gene expression arrays in MSCs after 24 h. A heatmap of cumulative values of fold expression represented by the minimum (red = 0) to the maximum (white = 300). Cumulative values; *N* = 3, *n* = 1.

The MSC transcriptome imparts cells with stemness characteristics. Therefore, this study evaluated the expression of stemness genes in MSCs cultured in proliferative and osteoinductive conditions to assess the effects of surface and culture medium on MSC fate determination. The three different donor-typed MSC populations used in this study displayed high variability in the expression of pluripotency markers. To circumvent patient-specific differences, cumulative fold values of genetic marker expression were determined by adding together the fold values obtained for each donor-typed MSC population. The results of this analysis are presented in a heatmap in Figure 6 with unexpressed genetic markers listed in Table 1 and cumulative fold values of expressed markers in Table 2.

The MSCs displayed medium- and surface-dependent trends in the expression of pluripotency markers. The three donor-specific MSCs similarly lacked expression of genetic markers listed in Table 1. These markers represent embryonic or extraembryonic cellular states. The cumulative fold values of gene expression appeared variably higher in MSCs cultured on the different Ti surfaces in osteogenic inductive medium compared to proliferative growth medium. The inference was that growth medium upregulated the “expression in undifferentiated cells/maintenance of pluripotency” and “correlation to stemness” related markers in MSCs on the modSLA along with differentiation markers compared to SLA and polished Ti. Conversely, osteogenic differentiation medium variably affected the expression of all three categories of pluripotency markers in addition to the endogenous controls in MSCs cultured on the different Ti surfaces. A variable downregulation in “correlation to stemness” and “differentiation” marker expression was observable in MSCs on both modified topographies compared to polished.

Table 2 indicates different gene transcription profiles for MSCs cultured on the SLA and modSLA Ti surfaces with the latter significantly increasing the expression of genes key to pluripotency, stemness, and differentiation in growth medium. These differences between MSCs on SLA and modSLA are largely omitted in osteogenic inductive medium with a higher trend of expression occurring on polished. Briefly, it is suggested that MSCs upregulate the expression of pluripotency markers on modSLA in proliferation inductive growth medium while osteogenic differentiation inductive medium reduces expression of pluripotency markers on both rough surfaces compared to polished Ti.

### 3.5. Osteogenic Mineralisation of MSCs on Different Surfaces

**Figure 7:**
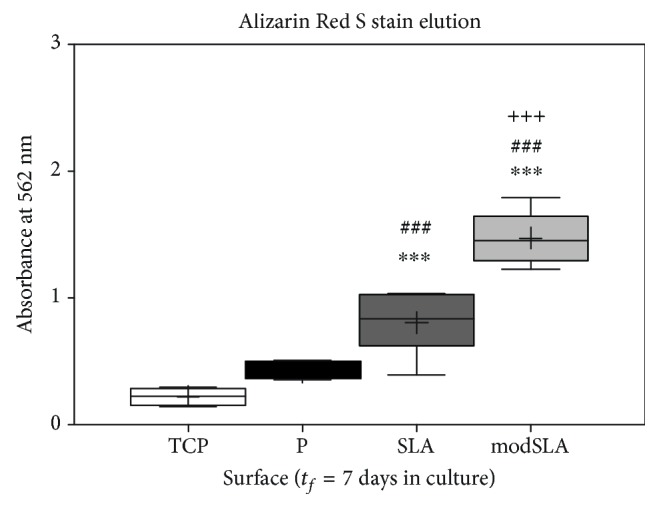
Assessment of osteogenic mineralisation with Alizarin Red S elution and spectrophotometry revealed rough Ti surfaces had induced significantly higher levels of calcium deposition by this time with the hydrophilic modSLA surface inducing significantly higher levels of calcium deposition than hydrophobic SLA. Box and whisker plot with minimum to maximum readings, *N* = 1; *n* = 3. ^∗∗∗^*p* < 0.001, TCP vs. any Ti. ^###^*p* < 0.001, P vs. rough. ^+++^*p* < 0.001, SLA vs. modSLA.

The results of this analysis are shown in Figure 7. Alizarin Red S stain retention was significantly higher for cells cultured on the modSLA surface (*p* < 0.001) compared to the other surfaces. The SLA cultured cells retained significantly more stain than polished Ti while the least stain was present on TCP.

## 4. Discussion

The present study has shown that in serum-free conditions, which negate the effects of deposited proteins and soluble factors, MSCs interact with Ti biomaterials to adhere remarkably early within very short contact durations and spread over a cross section of the substrate. The SLA and modSLA topographic modifications affect cellular adhesion and morphological adaptation causing a lesser number of cells to adhere. These adherent cells initially exhibit a highly spherical morphology while later adapting a stellate form. Conversely, MSCs adhere in larger numbers to polished Ti and exhibit an evenly concentric pattern of cellular spread while later adapting a stretched form. Gene expression analyses indicate a complex pattern of surface- and medium-dependent effects with Wnt signalling-associated Wnt5a, GDF15, MKI67, and BGLAP/OC expressed at higher levels in growth than osteoinductive medium after 24 hours of contact. The pluripotency miniarrays revealed increased stem cell-related gene expression in MSCs cultured in proliferative growth conditions on the hydrophilic modSLA compared to SLA and polished Ti, which contrasts with decreased gene expression in osteoinductive medium in MSCs on both modified surfaces than polished Ti. Osteogenic matrix mineralisation assessed at 7 days post cell surface contact was the highest on the modSLA and then the SLA compared to polished Ti and tissue culture polystyrene (TCP). Collectively, this complex matrix of data sets suggests that SLA and modSLA distinguish between different subsets of cells within the heterogeneous population of MSCs based on physical adaptability to micro- and nanotopographic modifications of the Ti substrates. Yet the most intriguing is the inference deduced by correlating these results with pluripotency marker-related gene expression in proliferative and osteoinductive conditions as well as osteogenic mineralisation that permits us to hypothesise that micro- and nanotopographic modifications of the modSLA anomalously influence increased genetic plasticity of adherent MSCs in instigating a novel form of MSC dedifferentiation or stemness.

Although a set of criteria are routinely applied to defining MSC populations [21], these commonalities are not sufficient in deciphering intercellular variability within a population of MSCs or between populations isolated from different tissues or between different donors [35]. Moreover, the criteria of clonogenicity, expression of specific cell surface markers, and trilineage differentiation do not represent a population of homogeneous multipotent progenitors rather a population that contains multipotent progenitors indecipherable from nonmultipotent cells based on current standards [35]. The process of selection observed in this study seemingly pertains to an ability of SLA and modSLA to distinguish multipotent progenitors from nonmultipotent cells based on cytoskeletal plasticity. This infers the ability of a subpopulation of MSCs to adhere to the modified topography earlier due to a larger capacity of modulating the cytoskeletal apparatus in conjunction with the activity of cell surface adhesion molecules.

The variability of cell surface responses to the modified Ti biomaterials appears to depend more on cytoskeletal reorganisation than the activity of cell surface adhesion molecules as inferred from the constrained morphology of a lesser number of cells on the rough compared to their evenly concentric spread in higher numbers on the polished. This implies that while adhesion is initiated by cell surface molecules, MSC cytoskeletal plasticity determines the continuation of adhesion, rate of cellular spread, and morphologic adaptation to the topography. Within the context of this study, this distinguishing ability of the rough topographies appears to lead to progenitors with higher cytoskeletal plasticity to adhere successfully and result in higher subsequent osteogenic mineralisation. The results also present an additional aspect of MSCs with higher cytoskeletal plasticity pertinent to genomic activity, which indicates that cells with higher cytoskeletal plasticity also have higher genomic plasticity compared to the heterogeneous population of MSCs adhering to polished Ti, discussed later in this section. While the extent of influence of nanotopography of the hydrophilic modSLA compared to the hydrophobic SLA on cellular selectivity could not be clearly delineated by the experiments conducted in this study, it can be confidently stated that the higher cellular adhesion seen on the polished Ti results in equal chances of adherence for subpopulations of varying cytoskeletal plasticity, resulting in the significantly lesser osteogenic mineralisation compared to the microrough topographies.

The pluripotency marker gene expression arrays were complicated by patient-specific differences even though the three adult MSC populations similarly unexpressed genes relevant to embryonic or fetal development. However, differences in cumulative fold expression between cells on the SLA and modSLA suggest differences in the population of cells adherent to both rough topographies, which is particularly evident in proliferation inductive growth medium. The suppression of gene expression by the rough surfaces in osteogenic inductive medium indicates the overriding potential of osteogenic differentiation cues from the immediate microenvironment in addition to the lesser effective cues of the polished Ti surface in inducing osteogenesis. Whilst this observation does not address why both rough surfaces have similar numbers of adherent cells yet seemingly significant differences in pluripotency marker expression, it essentially suggests an induction of dedifferentiation of adherent MSCs on the modSLA, which is observably contrasted in proliferative conditions and concurrent with the extent of osteogenesis in inductive conditions. Furthermore, the inclusion of microRNA analyses for phenotypic markers might have revealed a tight regulation of translation of molecules crucial to each of the differentiation pathways. This is a novel find that may delineate nanotopography of the modSLA to variably affect fate determination by instigating novel cell signalling pathways pertinent to stemness despite both topographies similarly constraining cellular adhesion and morphological adaption and effecting osteogenic mineralisation.

The phenomenon of dedifferentiation has been reported in various instances in the scientific literature such as [36]. It is definable as a reversible shift in the phenotype of cells induced through changes in experimental conditions [36], or a result of the natural process of repair [37] or an intricate aspect of the characteristics of cancer cells during tumorigenesis [38]. Our proposition here differs from these and other reports by inferring a change in the state of stemness of cells from the phenotype of culture-expanded MSCs to an earlier progenitorial state through the effect of a particular Ti biomaterial surface modification. While it would have been highly informative to have evaluated adherent and nonadherent cells with the pluripotency gene expression miniarrays as well as additional assays that may have further explained these observations, the very frugal yet complex matrix of experimentation provided here adequately supports the basis of these results and their interpretation. It is extremely serendipitous that these results link adult stem cell heterogeneity and parameters associated with the phenotype of stemness.

In conclusion, the comparison of MSC adhesion and morphological adaptation to rough Ti surfaces correlated to stemness-related gene expression presented here indicates the importance of early phenotypic events in conditioning cells to an enhanced osteogenic phenotype as well as the significance of topographical cues on cellular selection and multipotential differentiation. These results present a basis for the heterogeneity of MSCs by indicating cytoskeletal adaptability an important determinant of stem cell “age,” which seems to be directly related to genetic plasticity and is reversed by the micro- and nanostructured topography of the modSLA. Further elaboration of cellular and molecular processes underlying these cell-substrate interactions could improve implant surface characteristics to favourably adapt to weakened regenerative responses such as in the case of diabetes or age-related bone degenerative disorders. A consequence to this inference is that in addition to the current repertoire of cell surface markers used to immunophenotypically assess stem cell identity, as yet unidentified markers contributing to material-dependent fate determination of stem cells might further delineate cellular subsets within a heterogeneous MSC population.

## 5. Conclusion

This paper demonstrates that cytoskeletal plasticity is an intrinsic characteristic underlying the heterogeneity of a population of MSCs as shown by MSCs of a highly adaptable cytoskeleton overcoming the physical constraints of microstructure in adhering to rough topographic titanium biomaterials in the absence of deposited proteins or soluble factors. MSCs with higher cytoskeletal plasticity exhibit a higher level of genetic plasticity that is seemingly influenced by nanotopographic modifications in proliferative conditions and suppressed strongly by microtopographic modifications in osteoinductive conditions, thus underpinning the differential response of cells to the SLA and modSLA surfaces.

## Figures and Tables

**Table 1:** List of genetic markers not expressed in MSCs.

Gene symbol	Category	Gene symbol	Category
EEF1A1	Control	GCM1	Differentiation
TDGF1	Expression in undifferentiated cells	FOXA2	Differentiation
GDF3	Expression in undifferentiated cells	GATA4	Differentiation
SOX2	Maintenance of pluripotency	GCG	Differentiation
EBAF	Correlation to stemness	HBB	Differentiation
FGF4	Correlation to stemness	HBZ	Differentiation
FOXD3	Correlation to stemness	HLXB9	Differentiation
GAL	Correlation to stemness	IAPP	Differentiation
GBX2	Correlation to stemness	INS	Differentiation
LEFTB	Correlation to stemness	IPF1	Differentiation
LIN28	Correlation to stemness	ISL1	Differentiation
NODAL	Correlation to stemness	KRT1	Differentiation
NR5A2	Correlation to stemness	MYF5	Differentiation
PTEN	Correlation to stemness	MYOD1	Differentiation
SFRP2	Correlation to stemness	NEUROD1	Differentiation
TERT	Correlation to stemness	NPPA	Differentiation
TFCP2L1	Correlation to stemness	OLIG2	Differentiation
UTF1-	Correlation to stemness	PAX4	Differentiation
Xist	Correlation to stemness	PAX6	Differentiation
ZFP42	Correlation to stemness	PTF1A	Differentiation
AFP	Differentiation	SOX17	Differentiation
CD34	Differentiation	SYCP3	Differentiation
CDH5	Differentiation	T-	Differentiation
CDX2	Differentiation	TAT	Differentiation
COL2A1	Differentiation	TH	Differentiation
DDX4	Differentiation	WT1	Differentiation
EOMES	Differentiation		

**Table 2:** List of genetic markers and cumulative fold values of expression from three donors. Italicized values indicate the highest level of cumulative fold values of expression.

Gene symbol	GM P	GM SLA	GM modSLA	OM P	OM SLA	OM modSLA	Category
ACTB	*10.78*	2.90	0.86	26.47	*72.73*	2.77	Control
RAF1	2.41	1.77	*21.72*	*159.40*	17.47	39.55	Control
CTNNB1	5.43	3.77	*83.44*	*67.31*	33.16	41.68	Control
GAPD	2.43	2.98	*6.70*	1.40	6.34	*174.07*	Control
18S end control	3.00	3.00	3.00	3.00	3.00	3.00	Control
Nanog	6.81	2.62	*21.92*	4.29	*15.56*	3.91	Expression in undifferentiated cells/Maintenence of pluripotency
POU5F1	6.61	6.42	*259.53*	*235.38*	48.55	8.81	Expression in undifferentiated cells/Maintenence of pluripotency
DNMT3B	5.09	4.24	*116.44*	26.11	23.51	*38.12*	Expression in undifferentiated cells/Maintenence of pluripotency
GABRB3	5.77	5.31	*189.67*	*222.74*	10.99	126.86	Expression in undifferentiated cells/Maintenence of pluripotency
BRIX	4.79	1.50	3.88	3.03	6.01	*51.52*	Correlation to stemness
CD9	7.85	9.52	*687.18*	*70.21*	31.65	59.77	Correlation to stemness
COMMD3	3.95	3.89	*97.32*	*140.21*	32.07	103.13	Correlation to stemness
CRABP2	7.79	7.47	*42.14*	40.70	7.25	*58.92*	Correlation to stemness
FGF5	8.46	4.30	*36.90*	12.85	*21.94*	11.80	Correlation to stemness
GRB7	3.69	2.33	4.42	*21.31*	10.23	1.96	Correlation to stemness
IFITM1	11.03	17.96	*485.02*	5.04	14.71	*100.14*	Correlation to stemness
IFITM2	1.50	0.94	3.27	*2.71*	1.86	1.54	Correlation to stemness
IL6ST	5.48	4.24	*170.56*	*46.87*	19.47	20.64	Correlation to stemness
IMP2	1.88	1.68	3.53	4.41	6.93	*46.71*	Correlation to stemness
KIT	12.84	4.70	3.50	*36.20*	5.62	6.39	Correlation to stemness
LIFR	2.51	0.00	*18.35*	*401.85*	97.84	36.63	Correlation to stemness
NOG	0.87	0.42	*10.06*	*19.35*	3.53	4.46	Correlation to stemness
NR6A1	1.04	1.36	*4.34*	3.59	*6.10*	1.09	Correlation to stemness
PODXL	3.77	2.41	*91.91*	*22.15*	7.08	9.14	Correlation to stemness
REST	3.11	2.72	0.46	3.98	2.92	110.34	Correlation to stemness
SEMA3A	2.75	2.17	*38.41*	*33.00*	2.55	0.30	Correlation to stemness
ACTC	0.32	0.06	0.21	*1.32*	0.16	0.31	Differentiation
CGB	4.15	1.68	*10.72*	*25.19*	5.75	15.10	Differentiation
COL1A1	5.82	4.23	*112.68*	*16.47*	12.34	10.92	Differentiation
DES	5.58	*6.73*	3.49	24.31	*42.50*	25.70	Differentiation
FLT1	*23.27*	7.96	3.37	1.28	4.03	*5.75*	Differentiation
FN1	2.36	2.29	*27.37*	*28.44*	9.04	22.22	Differentiation
GATA6	3.40	2.63	*10.14*	*5.28*	2.28	4.11	Differentiation
GFAP	1.96	*2.20*	1.33	0.39	0.33	0.00	Differentiation
LAMA1	1.97	1.03	*32.05*	*133.14*	20.26	72.38	Differentiation
LAMB1	3.96	3.11	*28.62*	230.22	35.29	*233.72*	Differentiation
LAMC1	3.45	2.28	*31.04*	*43.53*	9.32	17.86	Differentiation
NES	3.00	1.45	14.82	*24.33*	3.67	6.19	Differentiation
PECAM1	18.22	19.32	*1133.63*	*824.73*	81.74	411.08	Differentiation
RUNX2	2.40	2.25	2.24	5.74	13.21	*56.92*	Differentiation
SERPINA1	20.17	20.92	*67.17*	11.01	*22.72*	5.60	Differentiation
SST	0.08	0.98	*7.81*	*1.82*	0.00	0.00	Differentiation
SYP	7.30	3.52	*67.06*	*182.41*	19.59	3.10	Differentiation

## Data Availability

The datasets generated during and/or analysed during the current study are available from the corresponding author on reasonable request.
